# Promoting early identification of sepsis in hospitalized patients with nurse-led protocols

**DOI:** 10.1186/s13054-016-1590-0

**Published:** 2017-01-11

**Authors:** Ruth Kleinpell

**Affiliations:** Rush University Medical Center, Chicago, Illinois USA

Nurses play a significant role in identifying patients with sepsis through their unique position of having constant patient interaction. As a result, sepsis screening can be integrated as part of routine patient assessments and patient care rounds [1]. A number of studies have established the impact of nurse-led sepsis screening interventions in improving early recognition of patients with sepsis.

In a study conducted in New Zealand, the “Sepsis Six” resuscitation bundle of care was used to raise awareness among staff and improve the management of patients with sepsis [2]. The bundle addressed care in six specific areas of sepsis care: intravenous fluids, blood cultures, antibiotics, lactate, oxygen, and urine output. Educational sessions were provided for nursing staff and junior doctors and algorithm posters served as visual reminders to implement the bundle, along with audit and feedback. The results demonstrated an improvement in the number of bundle measures that were implemented within 1 h, increasing from 29% pre-intervention to 63% post-intervention [2].

A retrospective analysis at a specialist oncology hospital in England was conducted after implementation of a nurse-led protocol for managing patients presenting with fever post-chemotherapy [3]; 672 (96.4%) patients presenting with suspected sepsis received their first dose of intravenous antibiotics within 60 min of presentation. Of this group, 323 (48.1%) were administered antibiotics within 15 min of arrival. The authors conclude that nurse-led protocols are an effective, safe, and sustainable method for achieving early antibiotic administration in patients with suspected febrile neutropenia [3].

In a novel rapid cycle process improvement initiative targeting early identification and treatment of sepsis, a “Sepsis Power Hour” was designated to meet the target of initiating elements of the sepsis bundle within 1 h of sepsis recognition: blood cultures checked, serum lactate checked, two liter isotonic fluid bolus started, and antibiotics started [4]. A protocol outlined the specific role of the nurse related to identification of sepsis, obtaining blood cultures and a lactate level and instituting a 500-cc fluid bolus. Bundle completion rates were compared to those of usual care patients with a random sample of 25 patients and the results demonstrated improvement in the time to starting a fluid bolus, obtaining a lactate level, and administering antibiotics [4].

Other studies on nurse-driven sepsis protocols have been shown to be highly effective in early identification and treatment of patients with sepsis. A nurse-driven care bundle-based sepsis protocol resulted in increased compliance with Surviving Sepsis Campaign [5] recommendations, including measuring serum lactate, obtaining two blood cultures before starting antibiotics, and starting antibiotics within 3 h, for patients presenting to the emergency department [6]. Similarly, implementation of a nurse-initiated sepsis protocol resulted in improved serum lactate measurement, blood culture collection, and median time to initial antibiotic administration in a tertiary academic medical center emergency department [7].

## Ward-based screening

Recently, studies focusing on ward-based nurse screenings for sepsis have also demonstrated benefit. A study from Norway targeted early identification of in-hospital sepsis by ward nurses [8]. As part of the Mid-Norway Sepsis Study, the study assessed the impact of a bundle intervention consisting of a flow chart for sepsis identification and physician notification and a clinical tool for triage of patients exhibiting signs of sepsis and organ failure. Additionally, a 4-h training course was provided to all nurses and nursing students working on the wards that included content on pathophysiology, signs of sepsis, and treatment recommendations, including the importance of fluid resuscitation, antibiotic therapy, and monitoring and communication of patient vital signs and condition status changes.

In comparison to a pre-intervention group of 472 patients with confirmed blood stream infection during a 2-year period, 409 patients with confirmed blood stream infection in a 2-year post-intervention period were found to have higher odds of surviving 30 days (odds ratio (OR) 2.7, 95% confidence interval (CI) 1.6–4.6), lower probability of developing severe organ failure (0.7, 95% CI 0.4–0.9), and, on average, 3.7 days (95% CI 1.5–5.9 days) shorter length of stay [8].

Another nurse-based early recognition and response program integrated an early sepsis screening tool into the electronic health record, screening and response protocols, and education and training of nurses with twice-daily screening of hospitalized patients and was found to be associated with reductions of inpatient sepsis-associated death rates [9]. These studies demonstrated significant differences not only in sepsis treatment but also length of stay and survival rates—positive outcomes that have not been consistently demonstrated in other studies of nurse-led screening or protocol use.

A recent multihospital quality improvement program focused on early detection and treatment of sepsis on general medical–surgical wards. Sixty sites engaged in a collaborative implementation process that used a basic screening tool and guidance for routine severe sepsis screening, monitoring, and feedback, and a structured scripted communication framework using the SBAR (situation, background, assessment, and recommendation) technique aimed to improve communication [10]. Key to the success of the initiative was an understanding that the training and experiences of ED, ICU, and ward nurses varies, necessitating that nurse education contain critical assessment skills to determine when to suspect a new or worsening infection.

The role of nurses in quality improvement of sepsis care is significant. As nurses spend the majority of time with patients, their role in the recognition and treatment of patients with sepsis is critical to improving sepsis-related outcomes [11, 12]. Educating all staff about sepsis management and the translation of guidelines into clinical practice can enhance the nurses’ ability to identify sepsis and implement early therapy measures [13]. Additionally, ensuring adequate education for nursing staff is a vital component to establishing highly functional sepsis screening and sepsis management protocols (Table 1).

**Table 1:** Key components of implementing nurse-led sepsis protocols

■ Use the international sepsis guidelines as a performance improvement initiative to identify gaps in care and specific areas for improvement. For example, track data related to sepsis care, including:
● Time to blood cultures
● Time to antibiotics
● Time to lactate levels
● Time to fluid bolus goals
● Compliance with all elements of the 3-h bundle
● Compliance with all elements of the 6-h bundle
■ Enlist administrative and physician stakeholder support to develop and pilot a nurse led sepsis protocol initiative
■ Provide a unit-, hospital-, and system-wide educational campaign that considers the varying level of nursing training and experience
■ Enlist nurse champions to spearhead the nurse-led protocol
■ Conduct ongoing data review and provide results to nursing staff and key stakeholders
■ Further refine processes based on ongoing audit data and feedback

Adapted from Kleinpell et al. [12]

Targeting early recognition of sepsis with use of multifaceted performance improvement initiatives has been demonstrated to improve compliance with sepsis performance measures with associated reductions in hospital mortality in patients with severe sepsis and septic shock in ICU and ward settings [8, 9, 14]. However, as sepsis remains a leading cause of mortality in critically ill patients worldwide, additional studies are needed to determine the most effective way to achieve sepsis bundle targets, including the incorporation of nurse-led screening and treatment protocols.
